# The obesity‐associated gene Negr1 regulates aspects of energy balance in rat hypothalamic areas

**DOI:** 10.14814/phy2.12083

**Published:** 2014-07-22

**Authors:** Arjen J. Boender, Margriet A. van Gestel, Keith M. Garner, Mieneke C. M. Luijendijk, Roger A. H. Adan

**Affiliations:** 1 Department of Translational Neuroscience, Brain Center Rudolf Magnus, University Medical Center Utrecht, Utrecht, The Netherlands

**Keywords:** AAV technology, arcuate nucleus, GWAS, Negr1, ventromedial hypothalamus

## Abstract

Neural growth regulator 1 (Negr1) is among the first common variants that have been associated with the regulation of body mass index. Using AAV technology directed to manipulate Negr1 expression in vivo, we find that decreased expression of Negr1 in periventricular hypothalamic areas leads to increases in body weight, presumably via increased food intake. Moreover, we observed that both increased and decreased levels of Negr1 lead to reduced locomotor activity and body temperature. In sum, our results provide further support for a role of hypothalamic expressed Negr1 in the regulation of energy balance.

## Abstract (precis)

Decreased expression of Negr1 in periventricular hypothalamic areas leads to increases in body weight, presumably via increased intake of carbohydrates. Moreover, we observed that both increased and decreased levels of Negr1 lead to reduced locomotor activity and body temperature. In sum, our results provide further support for a role of hypothalamic expressed Negr1 in the regulation of energy balance.

## Introduction

Neural growth regulator 1 (*Negr1*) – also known as kilon or neurotractin – is among the first common variants that have been associated with body mass index (BMI) (Thorleifsson et al. 2009; Willer et al. 2009; Speliotes et al. 2010), implicating *Negr1* in the etiology of obesity. Genome‐wide association studies (GWAS) in adults have shown that *Negr1* is associated with the regulation of BMI due to the existence of two distinct deletion alleles of 43 and 8 kb. Both deletions remove conserved, noncoding elements upstream of *Negr1*, but the 8‐kb deletion removes the binding site for NKX6.1 (Willer et al. 2009; Wheeler et al. 2013). As NKX6.1 is a strong transcriptional repressor, this suggests that altered expression of *Negr1* contributes to the regulation of BMI (Hafler et al. 2008).

As with most of the obesity‐associated genes, the function of *Negr1* in the etiology of obesity is yet to be determined. *Negr1* belongs to the IgLON group of proteins that serve as cell‐adhesion molecules and regulate cellular processes as neurite outgrowth and synapse formation (Hashimoto et al. 2008, 2009). IgLON members are expressed in different, but overlapping populations of neurons and can hetero‐ and homodimerize depending on cellular context (Lodge et al. 2000; McNamee et al. 2002). Changes in energy balance have been shown to influence synaptic organization and *Negr1* is a good candidate to be involved in this process (Pinto et al. 2004; Sternson et al. 2005). However, *Negr1* is also expressed on reactive astrocytes after experimental brain lesions, expanding its function to non‐neuronal cells as well (Schafer et al. 2005).

Previously, it was shown that *Negr1* is strongly expressed in the arcuate nucleus (ARC) and ventromedial hypothalamus (VMH) and is upregulated in these areas after food restriction (Boender et al. 2012). Moreover, mice that lack *Negr1* show decreased body weight and altered regulation of energy balance, suggesting that expression of *Negr1* indeed functions to regulate energy balance (Lee et al. 2012). We hypothesize that physiological regulation of expression of *Negr1* in the mediobasal hypothalamus is required to maintain energy balance upon exposure to dietary interventions. As systemic knockout of *Negr1* does not discriminate between developmental and direct effects on energy balance, we decided to use adeno‐associated viruses (AAV) to overexpress *Negr1* or to downregulate *Negr1* expression in the ARC/VMH of juvenile rats to dissect the direct affects of altered hypothalamic *Negr1* expression. In two separate experiments, we exposed AAV‐infected animals to a variety of diets to impose alterations in nutritional state. This allowed us to investigate the effects of altered hypothalamic *Negr1* expression on the regulation of energy balance in satiated and starved rats, thereby providing insight into the role of *Negr1* in the etiology of obesity.

## Material and Methods

### Animals and diet

Male wistar rats (*n* = 54) weighing 200–225 g on arrival were obtained from Charles‐River (Germany). All rats were individually housed in a controlled environment under a 12:12 h light/dark cycle, with lights on at 0700 h. During experiments 1 and 2, rats (*n* = 48) were exposed to different diets. The CHOW and refeeding (REF) diets consisted of ad libitum access to standard chow (Special Diet Service, Horley, UK), the high‐fat high‐sucrose diet (HFHS) consisted of ad libitum access to chow, saturated fat (Vandemoortele, Zeewolde, Belgium) and a 30% sucrose solution (Suiker Unie, Dinteloord, the Netherlands), while during the restriction diet (RFS), rats had access to chow for a 2‐h period each day starting at 1300 h. Food intake and body weight were determined on a regular basis. Experiments were approved by the Animal Ethics Committees of the University of Utrecht, according to Dutch legislation.

### Construction of plasmids

To obtain a NEGR1‐Renilla fusion plasmid, rat *Negr1* cDNA was amplified from hypothalamic rat cDNA. Primers were designed based on the published sequence (NM_021682.1) and contained attB‐sites to allow usage of the Gateway system (Invitrogen, Carlsbad, CA) for recombination cloning. *Negr1* cDNA was cloned into the Gateway entry vector pDONR201 (Invitrogen) and subsequently into a pBabe‐puro vector (Invitrogen) containing Renilla cDNA in order to get the NEGR1‐Renilla fusion plasmid.

The pAAV vector for inducing *Negr1* expression was synthesized from the pAAV‐CBA‐AgRP‐IRES‐GFP that has been described previously (de Backer et al. 2011). Briefly, *Negr1* cDNA was amplified from rat cDNA using polymerase chain reaction (PCR). Primers were designed using the published sequence of *Negr1* cDNA and contained BamHI recognition sites. With the use of BamHI restriction, *AgRP* cDNA was removed from pAAV‐CBA‐AGRP‐IRES‐GFP and *Negr1* cDNA was subsequently ligated into the linearized vector to obtain pAAV‐CBA‐NEGR1‐IRES‐GFP (pAAV‐NEGR1). Sequence analysis confirmed insertion of *Negr1* cDNA into the pAAV vector. This ligation was also performed in the absence of *Negr1* cDNA to obtain pAAV‐CBA‐IRES‐GFP (pAAV‐GFP).

For the generation of pAAVs‐expressing miRNAs, the Gateway system (Invitrogen) was used, as described in detail elsewhere (White et al. 2011). Briefly, the miRNA sequences for targeting *Negr1* were selected with “BLOCK‐iT^tm^ RNAi Designer” (http://rnaidesigner.lifetechnologies.com/rnaiexpress/) from Invitrogen (miR1: GTGCAGAGAACGATGTATCAT; miR2: GAGCACTTCGGCAACTATACT; miR‐luc: AAAGCAATTGTTCAGGAACC). The oligos covering these sequences were annealed and ligated into the synthetic intron region of PSM155 (Du et al.). The cassette containing the intronic miRNA upstream of EGFP was then amplified with B3 and B4 primers and recombined to generate the entry vectors pENTR‐R4‐miR1‐EGFP‐R3, pENTR‐R4‐miR2‐EGFP‐R3, and pENTR‐R4‐miRluc‐EGFP‐R3. To generate the pAAV‐ESYN‐miR1‐EGFP (pAAV‐miR1), pAAV‐ESYN‐miR2‐EGFP (pAAV‐miR2), and pAAV‐ESYN‐miRLuc‐EGFP (pAAV‐miRLuc), pENTR‐L1‐ESYN‐L4 and pENTR‐L3‐oPRE‐L2 were recombined with pAAV‐R1‐R2 and with either pENTR‐R4‐miR1‐EGFP‐R3, pENTR‐R4‐miR2‐EGFP‐R3, or pENTR‐R4‐miRluc‐EGFP‐R3.

### Luciferase assay

Human embryonic kidney (HEK) 293T cells were cultured in a 24‐well plate and were transfected using polyethylenimine (PEI) with 5 ng pcDNA4/TO‐luc, 500 ng pBabe‐NEGR1‐Renilla plasmid, and 500 ng pAAV‐miRs per well. Transfections were performed in quadruplo for each of the pAAV‐miRs. Two days after transfection, cells were lysed in passive lysis buffer and analyzed with a dual luciferase reporter assay according to the manufacturer's protocol (Promega, Madison, WI). Firefly and renilla luciferase activities were measured using a Viktor 96‐well plate reader (Perkin Elmer, Waltham, MA). Renilla luciferase activities were normalized to firefly luciferase activities and expressed as a percentage of the renilla luciferase activity after cotransfection of pAAV‐miRLuc.

### Procedures for viral infusion

Virus production was performed as described earlier (de Backer et al. 2010). All rats (*n* = 56) were bilaterally injected with 1.0 *μ*L of 1.0 × 10^9^ genomic copies/*μ*L of AAV in the ARC/VMH (from bregma: anterio‐posterior: −2.6 mm, medio‐lateral: ±1.2 mm, dorso‐ventral: −9.7 mm, at an angle of 5°). In experiment 1, rats (*n* = 24) were injected with AAV‐miR1, AAV‐NEGR1, AAV‐GFP, or AAV‐miRLuc; in experiment 2, rats (*n* = 24) were bilaterally injected with 1.0 *μ*L of 1.0 × 10^9^ genomic copies/*μ*L of either AAV‐miR1, AAV‐miR2, or AAV‐miRLuc; and in experiment 3, rats (*n* = 8) were unilaterally injected with AAV‐miRLuc and received contralateral infusions of either AAV‐miR1 or AAV‐miR2 in the ARC/VMH. Infusions were performed under fentanyl/fluanisone (0.315 mg/kg fentanyl, 10 mg/kg fluanisone, i.m., Hypnorm, Janssen Pharmaceutica, Beerse, Belgium) and midazolam (2.5 mg/kg, i.p., Actavis, Baarn, the Netherlands) anesthesia. Xylocaine was sprayed on the skull to provide local anesthesia (Lidocaine 100 mg/mL, AstraZeneca BV, Zoetermeer, the Netherlands). To reduce pain symptoms, all rats received three daily injections of carprofen (5 mg/kg, s.c.) starting at the day of surgery. In addition, rats in experiment 1 received a transmitter for the recording of locomotor activity and body temperature (TA10TA‐F40, Data Science International, St. Paul, MN, USA) in the abdominal cavity.

#### Experiment 1

Baseline measurements of body weight and food intake were taken in the week before surgery to divide animals into four experimental groups (AAV‐NEGR1 [*n* = 7], AAV‐miR1 [*n* = 7], AAV‐GFP [*n* = 4], and AAV‐miRLuc [*n* = 3]) that were equal in body weight and food intake. As AAV‐GFP and AAV‐miRLuc animals did not differ in body weight or food intake throughout the experiment (data not shown), the groups were combined to AAV‐CTRL. During the first 10 weeks after surgery, rats were exposed to the CHOW diet. Subsequently, rats were exposed to the RFS diet for 8 days in order to determine whether interference with *Negr1* expression affected the response to food restriction. Following 8 days of food restriction, rats were allowed to regain their body weight on the REF diet for a period of 2 weeks. Locomotor activity and body temperature were determined in week 5 after surgery by placing the home cage on a receiver plate (DSI) that received radiofrequency signals from the abdominal transmitter. The plate was connected to software (DSI) that recorded the locomotor activity within 600 sec bins. Body temperature was determined at the end of each 600 sec bin. Locomotor activity and body temperature were determined during 7 days and averaged over light and dark phases. After completion of the experiments, rats were decapitated. Their brains were carefully dissected out, quickly frozen on dry ice, and stored at −80°C.

#### Experiment 2

Baseline measurements of body weight and food intake were taken in the week before surgery to divide animals into three experimental groups (AAV‐miR1 [*n* = 7], AAV‐miR2 [*n* = 8], and AAV‐miRLuc [*n* = 8]) that were equal in body weight and food intake. During the first 3 weeks after surgery, rats were exposed to the CHOW diet. Subsequently, rats were food restricted for 6 days (RFS) in order to determine whether interference with *Negr1* expression affected the response to food restriction. Following food restriction rats were allowed to regain their body weight for a period of 2 weeks under exposure to the REF. Finally, rats were exposed to the HFHS diet for 1 week in order to determine whether interference with *Negr1* expression affected the response to increased caloric intake. After completion of the experiments, rats were decapitated. Their brains were carefully dissected out, quickly frozen on dry ice, and stored at −80°C.

#### Experiment 3

For this experiment rats were kept on chow. Baseline measurements of body weight and food intake were taken in the week before surgery to divide animals into two experimental groups, AAV‐miR1 (*n* = 4) and AAV‐miR2 (*n* = 4), which were equal in body weight and food intake. Six weeks after surgery animals were decapitated, their brains were carefully dissected out, quickly frozen on dry ice, and stored at −80°C. These animals were used to determine the in vivo knockdown efficiency of AAV‐miR1 and AAV‐miR2 (as described below).

### Digoxigenin, LNA, and ^33^P in situ hybridization procedures

Digoxigenin, LNA, and ^33^P in situ hybridization procedures were performed as described previously (Obernosterer et al. 2007; Tiesjema et al. 2007; de Backer et al. 2010). Riboprobes used DIG‐labeled riboprobe against GFP mRNA (DQ768212), LNA probes against miR‐124 were purchased from Exiqon (Denmark), and ^33^P‐labeled riboprobe against *Negr1* (NM_021682.1, pos: 144–699). Correct targeting of AAVs in the hypothalamus was done in all animals used in experiments 1 and 2 by means of DIG‐ISH against GFP mRNA. Validation of AAV‐NEGR1 was done in four animals used in experiment 2 by means of a ^33^P‐ISH against *Negr1* mRNA.

### In vivo knockdown efficiency

To check if AAV‐miRs did knockdown expression of *Negr1* in the ARC/VMH, these areas were microdissected from five 300 *μ*m coronal sections that were cut on a cryostat (Leica, Mannheim, Germany) and combined samples from each hemisphere were collected in TRIzol Reagent. RNA was isolated following manufacturer's instructions (Invitrogen) and the concentration and quality was determined using a spectrophotometer (Qiagen, Valencia, CA) and 1% agarose gel electrophoresis. To determine levels of ARC/VMH *Negr1* expression, one‐step qPCR was performed using a Quantifast SYBR Green RT‐PCR kit (Qiagen) and a LightCycler (Roche, Mannheim, Germany), according to manufacturer's instruction. To determine knockdown efficiency of AAV‐miR1 and AAV‐miR2 different primer sets were used, because care was take to span the target sites of the different miRs (for AAV‐miR1: FW: TCTCCCCATCAGCAAAACCA, RV: CGCAAAGTTCACGACCACTC and for AAV‐miR2: FW: GTGACACAGGAGCACTTCGG, RV: GTGCTTGGAGGGTTGAGGGG). Cycling conditions were 15 min at 50°C, 10 min at 95°C, followed by 35 cycles of 15 sec at 95°C, 30 sec at 60°C, and 30 sec at 72°C. After cycling, a melting protocol was performed, from 60 to 95°C, measuring fluorescence every 1°C, to control for product specificity. *Negr1* expression was first normalized to the expression of the household genes (Actb: FW: CGTGAAAAGATGACCCAGATCA, RV: AGAGGCATACAGGGACAACACA and CycA: FW: AGCACTGGGGAGAAAGGATT, RV: AGCCACTCAGTCTTGGCAGT). Next, the normalized *Negr1* expression on the side with AAV‐miR1 or AAV‐mir2 was expressed as percentage of expression of *Negr1* on the side with AAV‐miRLuc.

### Statistical analyses

All statistical analyses were performed using SPSS 20 for Windows (IBM, Armonk, NY). Thresholds of significance were set at *α *≤ 0.05.

## Results

### Knockdown efficiency of pAAV‐miR1 and pAAV‐miR2

**Figure 1.**
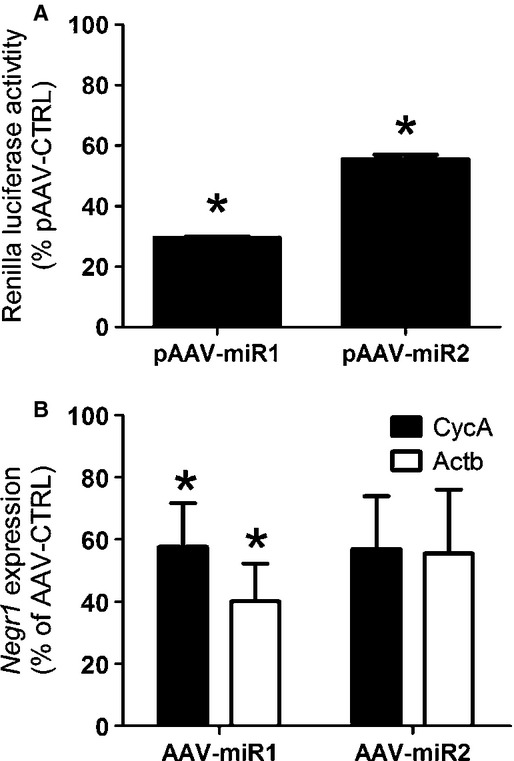
Knockdown efficiency of pAAV‐miRs. (A) Bars depict the mean normalized percentages (+SEM) of the renilla luciferase activity of the NEGR1‐renilla fusion plasmid after transfection of pAAV‐miR1 and pAAV‐miR2. *A significant difference between pAAV‐CTRL and pAAV‐miR1 or pAAV‐miR2 (*P* ≤ 0.05). (B) Bars depict the mean normalized percentages to either *CycA* or *Actb* expression (+SEM) of Negr1 expression after unilateral infusion of pAAV‐miR1 and pAAV‐miR2. *A significant difference between AAV‐CTRL and AAV‐miR1 or AAV‐miR2 (*P* ≤ 0.05).

To determine the in vitro knockdown efficiency of both pAAV‐miRs, a dual luciferase assay was employed. Both cotransfection of pAAV‐miR1 or pAAV‐miR2 with the NEGR‐renilla fusion plasmid led to significant knockdown, when compared to cotransfection with pAAV‐miRLuc. Both pAAV‐miR1 (one sample *t*‐test, *t* = −151.062, *P* < 0.001) and pAAV‐miR2 (one sample *t*‐test, *t* = −21.578, *P* < 0.001) successfully decreased *Negr1* expression in vitro (Fig. 1A). To examine in vivo knockdown efficiency, rats were bilaterally injected in the ARC/VMH with AAV‐miR1 or AAV‐miR2 on one side and AAV‐CTRL on the other side. Infusion of AAV‐miR1 (one sample *t*‐test, *t* ≤ 3.289, *P* ≤ 0.030), but not AAV‐miR2 (*t* ≥ 2.521, *P* ≥ 0.086) led to a significant decrease in ARC/VMH expression (Fig. 1B).

### Validation of the infusion sites and miR‐124 expression

**Figure 2.**
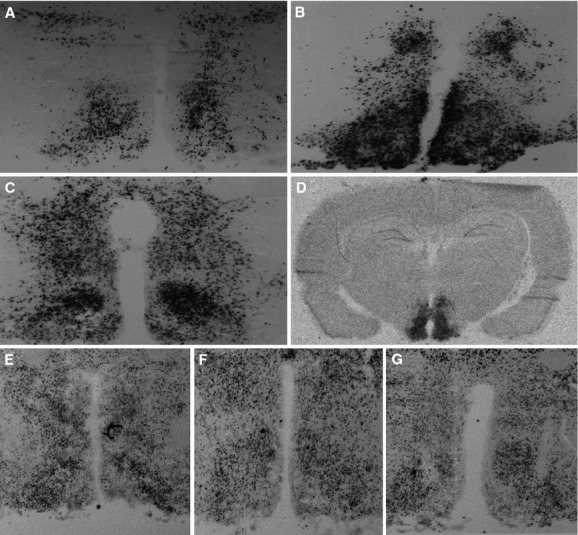
Validation of the infusion sites and miR‐124 expression. (A–C) Infusion of all AAVs led to GFP expression in the ARC/VMH, indicating that infusions were placed in the right areas. (A) Example of GFP expression after AAV‐CTRL infusion. (B) Example of GFP expression after AAV‐miR1 infusion. (C) Example of GFP expression after AAV‐miR2 infusion. (D) Infusion of AAV‐NEGR1 led to increased expression of Negr1 in the ARC/VMH. (E–G) Infusion of AAV‐miRs did not lead to significant cell death, as evidenced by miR‐124 expression, which was equal after infusion of AAV‐CTRL (E), AAV‐miR1 (F), and AAV‐miR2 (G).

Infusion of all AAVs led to expression of GFP mRNA in the ARC/VMH (Fig. 2A–C), indicating that infusions were placed in the right area. In addition, infusion of AAV‐NEGR1 led to increased expression of *Negr1* (Fig. 2D). Moreover, miR‐124 expression was not altered between AAV‐CTRL and AAV‐miR animals (Fig. 2E–G), indicating that AAV‐miRs did not disturb processing of endogenous miRNA in the ARC/VMH, as miR‐124 expression serves as a proxy for neuronal cell viability due to oversaturation of the endogenous miRNA processing machinery (van Gestel et al. 2014).

### Effect of AAV‐miR1 and AAV‐NEGR1 infusion in the ARC/VMH on body weight, food intake, locomotor activity, and body temperature under CHOW exposure

**Figure 3.**
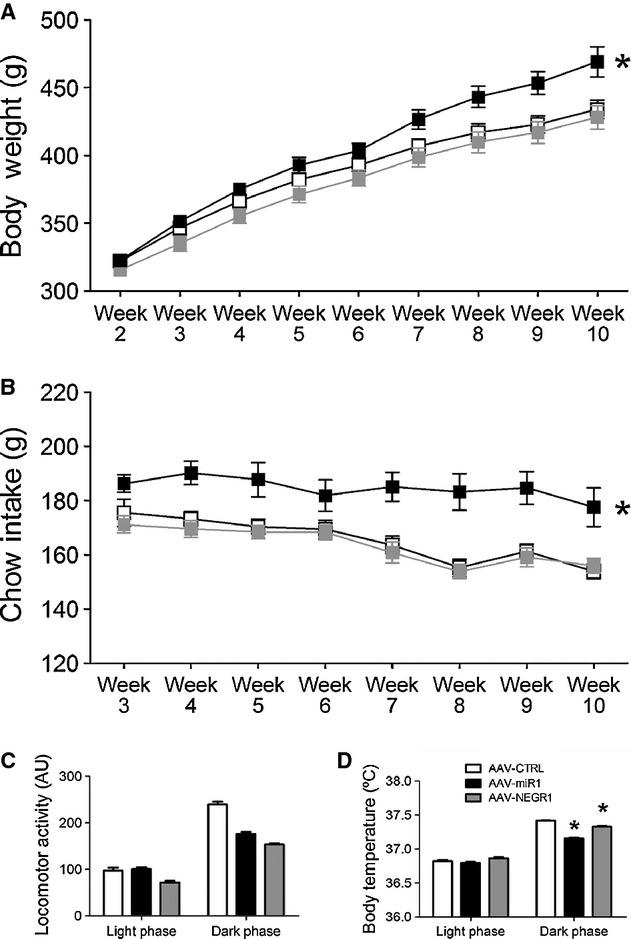
Effect of AAV‐miR1 and AAV‐NEGR1 infusion in the ARC/VMH on body weight, food intake, locomotor activity, and body temperature during CHOW exposure. Depicted are the mean (±SEM) body weight (A) and weekly chow intake (B) in the weeks after surgery of AAV‐miR1, AAV‐NEGR1, and AAV‐CTRL animals. *A significant difference between AAV‐miR1 and AAV‐CTRL (*P* ≤ 0.05). Bars depict the mean (+SEM) daily locomotor activity (C) and body temperature (D) during the light or dark phase. *A significant difference between AAV‐CTRL and AAV‐miR1 or AAV‐NEGR1 animals (*P* ≤ 0.05).

To determine whether altered expression of *Negr1* affects energy balance, AAV‐NEGR1 and AAV‐miR1 were infused bilaterally into the ARC/VMH of rats to increase or decrease *Negr1* expression, respectively. To ensure effectiveness of AAV vectors, measurements started 2 weeks after surgery. Under CHOW exposure, there was an effect of AAV treatment on body weight (repeated measures ANOVA, *f* = 5.387, *P* = 0.015) and food intake (repeated measures ANOVA, *f* = 11.272, *P* = 0.001). AAV‐miR1 animals showed increased body weight (LSD post hoc, *P* = 0.043) and increased food intake (LSD post hoc, *P* = 0.001), while AAV‐NEGR1 animals did not significantly differ from AAV‐CTRL in body weight (LSD post hoc, *P* = 0.311) or food intake (LSD post hoc, *P* = 0.698) (Fig. 3A and B). AAV treatment did also affect locomotor activity (univariate ANOVA, *f* = 76.821, *P* < 0.001). AAV treatment showed an interaction with light/dark phase (*f* = 32.583, *P* < 0.001) and subsequent analyses showed that both AAV‐miR1 and AAV‐NEGR1 animals were less active than AAV‐CTRL animals during the dark phase (one way ANOVA, *f* = 103.416, *P* < 0.001, LSD post hoc, *P* ≤ 0.001), but that only AAV‐NEGR1 animals showed decreased activity during the light phase (LSD post hoc, *P* < 0.001) (Fig. 3C). In addition, AAV treatment did affect body temperature (univariate ANOVA, *f* = 78.567, *P* < 0.001). As with locomotor activity, AAV treatment showed an interaction with light/dark phase (*f* = 46.504, *P* < 0.001) and subsequent analyses indicated that both AAV‐miR1 and AAV‐NEGR1 animals showed decreased body temperature during the dark phase (ANOVA, *f* = 231.000, *P* < 0.001, LSD post hoc *P* < 0.001), but that AAV‐miR1, AAV‐NEGR1, and AAV‐CTRL animals showed comparable body temperatures during the light phase (Fig. 3D).

### Effect of AAV‐NEGR1 on body weight and food intake under RFS and REF exposure

**Figure 4.**
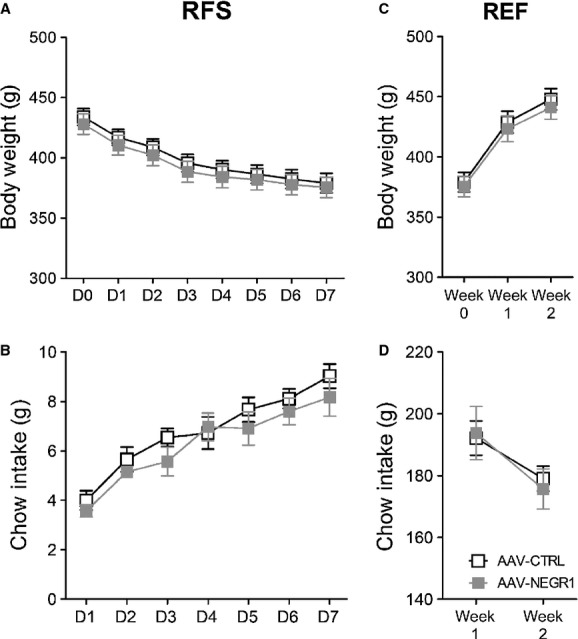
Effect of AAV‐NEGR1 infusion in the ARC/VMH on body weight and food intake during RFS and REF exposure. Depicted are the mean (±SEM) body weight (A) and daily chow intake (B) of AAV‐NEGR1 and AAV‐CTRL animals under exposure to food restriction (RFS). Depicted are the mean (±SEM) body weight (C) and weekly chow intake (D) of AAV‐NEGR1 and AAV‐CTRL animals under exposure of the refeeding diet (REF).

Since AAV‐NEGR1 animals did not differ in body weight or food intake from AAV‐CTRL animals, we continued experiments with AAV‐CTRL and AAV‐NEGR1 animals. After chow exposure, AAV‐CTRL and AAV‐NEGR1 animals were subjected to food restriction (RFS) for 1 week. Under RFS exposure, there was no main effect of AAV type on body weight (repeated measures ANOVA, *f* = 0.246, *P* = 0.629) or food intake (repeated measures ANOVA, *f* = 0.878, *P* = 0.367) (Fig. 4A and B). Then, AAV‐NEGR1 and AAV‐CTRL animals were allowed to refeed on ad libitum chow for 2 weeks (REF). Under REF exposure, there was no effect of AAV‐NEGR1 on body weight (repeated measures ANOVA, *f* = 0.217, *P* = 0.650) or food intake (repeated measures ANOVA, *f* = 0.117, *P* = 0.738) (Fig. 4C and D).

### Effect of AAV‐miR1 and AAV‐miR2 infusions in the ARC/VMH on body weight and food intake under exposure to restricted feeding, refeeding, and high‐energy diets

**Figure 5.**
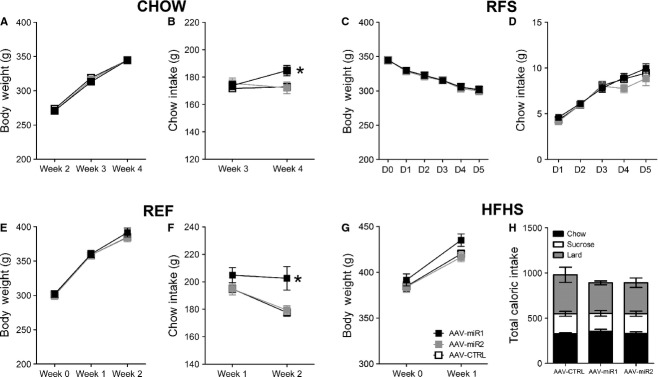
Effect of AAV‐miR infusion in the ARC/VMH on body weight and food intake during CHOW, RFS, REF, and HFHS exposure. Depicted are the mean (±SEM) body weight (A) and daily chow intake (B) of AAV‐miR1, AAV‐miR2, and AAV‐CTRL animals under chow exposure (CHOW). Depicted are the mean (±SEM) body weight (C) and weekly chow intake (D) of AAV‐miR1, AAV‐miR2 and AAV‐CTRL animals under exposure to food restriction (RFS). Depicted are the mean (±SEM) body weight (E) and weekly chow intake (F) of AAV‐miR1, AAV‐miR2, and AAV‐CTRL animals under exposure to the refeeding diet (REF). (G) Depicted are the mean (±SEM) body weight (E) of AAV‐miR1, AAV‐miR2, and AAV‐CTRL animals under exposure to the high‐fat high‐sucrose diet (HFHS). (H) Bars represent the mean weekly intake of chow, lard and sucrose in calories (±SEM) of AAV‐miR1, AAV‐miR2, and AAV‐CTRL animals. *A significant difference between AAV‐CTRL and AAV‐miR1 animals (*P* ≤ 0.05).

In a second experiment, we investigated the effect of AAV‐miR1 and miR2 infusions in the ARC/VMH to replicate and extend on the previous findings that AAV‐miR1 infusion in the ARC/VMH affects energy balance. As stated earlier, there was a significant effect of AAV treatment on food intake (repeated measures ANOVA, *f* = 4.282, *P* = 0.028), as AAV‐miR1 did show significantly increased food intake (LSD post hoc, *P* = 0.021), while AAV‐miR2 animals did not show differences in food intake when compared to AAV‐CTRL animals (LSD post hoc, *P* = 0.899) (Fig. 5B). No differences in body weight between AAV‐miR animals and AAV‐CTRL animals could be observed (repeated measures ANOVA, *f* = 0.001, *P* = 0.999) (Fig. 5A), which allowed for unbiased investigation of the effect of the AAV‐miRs on energy balance under RFS exposure. However, no differences in body weight (repeated measures ANOVA, *f* = 0.029, *P* = 0.971) or food intake could be observed under RFS exposure (repeated measures ANOVA, *f* = 0.721, *P* = 0.498) (Fig. 5C and D). Next, the animals were exposed to the REF diet. Again, no differences could be observed in body weight during the 2 weeks of REF exposure between AAV‐miR and AAV‐Negr1 animals (repeated measures ANOVA, *f* = 0.158, *P* = 0.855), although there was an effect of AAV treatment on food intake (repeated measures ANOVA, *f* = 4.362, *P* = 0.027) (Fig. 5E and F), as AAV‐miR1 animals did show significantly increased chow intake (LSD post hoc, *P* = 0.026). Finally, all animals were subjected to HFHS exposure for 1 week. HFHS exposure did not lead to significant differences in body weight (one way ANOVA, *f* = 3.205, *P* = 0.062), chow intake (one way ANOVA, *f* = 0.853, *P* = 0.441), sucrose intake (one way ANOVA, *f* = 0.206, *P* = 0.815), or lard intake (one way ANOVA, *f* = 0.829, *P* = 0.451) (Fig. 5G and H).

## Discussion

In this study, we used AAV technology to increase and knockdown expression of the obesity‐associated gene *Negr1* in the ARC/VMH and determine subsequent effects on the regulation of energy balance. Before discussion of the results, it must be noted that the AAVs did not only target the ARC/VMH but also in some rats spread to part of the dorso‐medial hypothalamus (DMH) as well. Therefore, it cannot be concluded that the observed effects were mediated by the ARC/VMH only, especially because both areas regulate energy balance. Moreover, we used two AAV‐miRs to knockdown *Negr1* expression, each targeting different exons within the *Negr1* sequence. Both AAV‐miRs did show effective knockdown in vitro, but only AAV‐miR1 achieved significant knockdown in vivo. Since not all *Negr1*‐expressing cells may have been transduced, demonstration of effective knockdown in vivo is not a measure of average knockdown in *Negr1*‐expressing cells, but reflecting knockdown in transduced cells among all *Negr1*‐expressing cells in the dissected brain area. AAV‐miR2 did reach borderline significance for knockdown in vivo and showed comparable percentages of knockdown when compared to AAV‐miR1. This complicates interpretation of the results, in particular because AAV‐miR2 did not have any effect on the regulation of energy balance. As AAV‐miR2 did not induce sufficient knockdown of *Negr1* to influence the regulation of energy balance further discussion will be limited to the results of AAV‐miR1.

Decreased expression of *Negr1* led to increases in chow intake under ad libitum chow exposure (CHOW and REF), resulting in increased body weight in case of prolonged exposure, while increased expression of *Negr1* did not affect any of these parameters. During exposure to food restriction or to the high‐fat high‐sucrose diet, no effect of altered *Negr1* expression could be observed on body weight or energy intake. Two independent studies have found an association of *Negr1* with increases in carbohydrate and fiber intake in the human population (Bauer et al. 2009; Rukh et al. 2013). One interpretation for our results therefore is that decreased *Negr1* expression selectively increases the intake of carbohydrates, the main macronutrient in standard laboratory chow. However, AAV‐miR animals did not consume more sucrose upon exposure to the HFHS diet (Fig. 5H). In addition, *Negr1* has been associated with decreases in fat intake (Bauer et al. 2009). Although the effect of decreased *Negr1* expression on fat intake was not significant, AAV‐miR animals did eat less fat than AAV‐CTRL animals. Prolonged exposure to the high‐fat high‐sucrose diet and other experiments directed to investigate the effect of *Negr1* on food choice could clarify if decreased expression of *Negr1* indeed selectively alters the intake of different macronutrients.

Both increases in and knockdown of *Negr1* expression did lead to decreases in locomotor activity and body temperature. Although this effect might be counterintuitive, an explanation can be found in earlier results from our laboratory (Boender et al. 2012). We showed that *Negr1* expression in the ARC/VMH is increased after periods of food restriction, which could reflect an adaptive response directed to preserve energy. Although decreased *Negr1* expression also decreased locomotor activity and body temperature, this effect might have been secondary to the effect of increased energy intake, in an attempt to compensate for a positive energy balance.

Finally, our results are not congruent with a recent study that reported the effects of a systemic *Negr1* knockout in mice (Lee et al. 2012). These mice showed decreases in body weight, food intake, and locomotor activity. Species differences and developmental effects could account for these phenotypical differences. Moreover, it should be noted here that the deletion alleles near *Negr1* associate with an increased BMI (Willer et al. 2009; Wheeler et al. 2013), suggesting that decreased expression of *Negr1* leads to increased body weight, which is consistent with our observations. At any rate, the combined results of these studies point toward a significant role for *Negr1* in the regulation of energy balance, possibly by controlling the intake of specific macronutrients and the regulation of energy expenditure.

## Conflict of Interest

None declared.
